# Holter study of heart rate variability in children and adolescents with long QT syndrome

**DOI:** 10.1111/anec.13132

**Published:** 2024-06-18

**Authors:** Anna Lundström, Håkan Eliasson, Marcus Karlsson, Urban Wiklund, Annika Rydberg

**Affiliations:** ^1^ Department of Clinical Sciences Umeå University Umeå Sweden; ^2^ Department of Women's and Children's Health Karolinska Institutet Stockholm Sweden; ^3^ Department of Diagnostics and Intervention, Biomedical Engineering and Radiation Physics Umeå University Umeå Sweden

**Keywords:** 24‐h electrocardiogram, beta‐blocker, heart rate variability, Holter, long QT syndrome, pediatric

## Abstract

**Objectives:**

This study aimed to retrospectively assess cardiac autonomic activity in children with LQTS, considering genotype, symptoms, sex, age, and beta‐blocker therapy (BB) and compare it to healthy controls.

**Methods:**

Heart rate variability (HRV), using power spectrum analysis, was analyzed in 575 Holter recordings from 116 children with LQTS and in 69 healthy children. The data were categorized into four age‐groups and four heart rate (HR) ranges.

**Results:**

In LQT1 and LQT2, increasing HR corresponded to significantly lower low (LF) and high frequency (HF) compared to controls. Total power (PTOT) was lower in all LQT1 age‐groups compared to controls at HR 120–140 bpm (1–15 years: *p* < .01; 15–18 years: *p* = .03). At HR 80–100, LQT1 patients aged 1–10 years had lower HF than LQT2 patients (1–5 years: *p* = .05; 5–10 years: *p* = .02), and LQT2 patients aged 15–18 years had lower HF than LQT1 patients (*p* < .01). Symptomatic patients aged 10–15 years had lower PTOT at HR 100–120 bpm than asymptomatic patients (*p* = .04). LQT1 girls aged 10–15 and 15–18 years had a lower PTOT (10–15 years: *p* = .04; 15–18 years: *p* = .02) than boys.

**Conclusion:**

This study shows a correlation between HR and changes in HRV parameters. At higher HRs, LQTS patients generally had lower HRV values than controls, suggesting an abnormal autonomic response. These results may strengthen the link between physical activity and arrhythmias in LQTS.

## Graphical abstract

This study compared cardiac autonomic activity in 116 LQTS children to 69 healthy controls using 24‐h ECG recordings. Lower heart rate variability (HRV) values in LQTS patients were found at higher heart rates (HRs), indicating an abnormal autonomic response. This may strengthen the link between LQTS, physical activity, and arrhythmias.

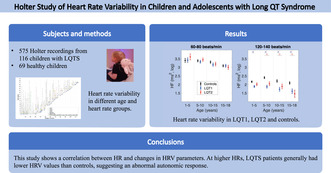

## 1 INTRODUCTION

Over the past few decades, extensive research has been conducted on genotype–phenotype correlations in inherited arrhythmic diseases. Hereditary long QT syndrome (LQTS) is a disease that poses a life‐threatening risk due to arrhythmias caused by mutations in genes that encode cardiac ion channels. Although the phenotypes are comparable, with prolonged QT intervals, syncope, and sudden death, the underlying trigger mechanisms vary. Therefore, clinicians must consider the underlying genotype causing the disease to manage and treat patients effectively (Zareba et al., 1998). In long QT syndrome type 1 (LQT1), sudden increased sympathetic activity, often triggered by physical or mental stress, is associated with arrhythmia (Schwartz et al., 2001). For LQT2 patients, the trigger is often an auditory stimulus, especially upon awakening (Schwartz et al., 2001). LQT3 patients predominantly experience cardiac events during sleep, bradycardia, or intermittent atrioventricular (AV)‐block, indicating increased parasympathetic predominance (Zareba et al., 1998). Correspondingly, all these triggers are due to fluctuations in the autonomic nervous system (ANS). This, together with the fact that beta‐adrenergic blocking and left cardiac denervation are effective treatments for LQTS, highlights the involvement of the ANS in this disease (Moss et al., 2000). It also underscores the importance of understanding the interaction between the sympathetic and parasympathetic nervous systems in LQTS.

One of the most commonly used markers of autonomic function is heart rate variability (HRV). This is a non‐invasive technique that assesses the impact of the ANS modulation on the cardiac sinus node. However, there is a lack of conclusive and consistent data in studies on HRV in LQTS patients. Previous research has shown HRV abnormalities in adult LQTS patients (Morita et al., 1996; Porta et al., 2015; Shamsuzzaman, 2003), but there have also been reports of normal HRV findings (Perkiömäki et al., 2001). Nevertheless, there remains limited knowledge about how HRV changes with age in children and adolescents with LQTS.

Age has been acknowledged to influence several clinical aspects in LQTS patients. For example, is the QTc interval influenced by age, sex, and genotype (Vink et al., 2017, 2018). Additionally, age is known to influence the risk of cardiac events and patients with LQTS often experience their first cardiac event before the age of 16 (Zareba et al., 1995). Furthermore, a sex‐related age difference at onset of cardiac events in LQT1 patients has also been found, where males tend to have a higher risk before puberty than females (Hobbs et al., 2006; Locati et al., 1998). A difference in risk assessment between LQT2 males and females has also been found, where adult females with LQT2 face a significantly higher risk compared to their male counterparts (Zareba et al., 2003). This makes it even more intriguing to investigate potential age‐, sex‐, and genotype‐related variations of HRV in children with LQTS.

Since assessing the individual risk of sudden cardiac events and death is a significant challenge in managing LQTS patients, objective parameters and additional indicators are necessary to evaluate both the risk of events and the efficacy of medical treatment. Twenty‐four‐hour Holter electrocardiogram (ECG) recordings are used clinically in LQTS patients to detect arrhythmias and evaluate medical compliance. From these recordings, HRV can be assessed, and this enables examination of HRV responses during daily activities and sleep.

To the best of our knowledge, the present study is the first to focus on HRV in Holter recordings in a pediatric LQTS population. The aim of this retrospective study was to investigate cardiac autonomic activity by evaluating HRV in regularly performed Holter recordings in children and adolescents with LQTS and healthy controls.

## 2 METHODS

### 2.1 Study population

In this retrospective study, a total of 118 patients between the ages of 1 and 18 years with genetically confirmed LQT1, LQT2, LQT3, and Jervell and Lange‐Nielsen syndrome (JLNS) were included. All patients underwent at least one standard 24‐h ambulatory Holter ECG recording between the year 2000 and 2021. Two subjects were excluded from the study due to inadequate recording quality. As a result, the final study population consisted of 116 subjects and a total of 575 Holter recordings. The median age at the time of the recording was 7.5 years. Nine patients had a pacemaker or an implanted cardioverter defibrillator (LQT1: *n* = 2, LQT2: *n* = 1, LQT3: *n* = 2, JLNS: *n* = 4), but sequences with paced beats were excluded from the analysis of HRV. Symptomatic patients were defined as patients who had experienced cardiac events such as syncope or aborted cardiac arrest. The patients attended regular follow‐ups at either the Department of Pediatric Cardiology at Norrland's University Hospital, Umeå, or Astrid Lindgren Children's Hospital, Karolinska Hospital, Solna, Sweden.

The control group consisted of at least one female and one male of each annual interval between 1 and 18 years (34 females, 35 males), totally 69 healthy children and adolescents. The median age was 10.4 years (ranging 1 to 18 years). They were recruited in Umeå through local advertisements and underwent Holter monitoring and echocardiography. All control subjects showed normal findings.

### 2.2 Data collection

The medical records of each patient were reviewed retrospectively, to gather data on LQTS genotype, sex, age, height, weight, and symptoms and whether they were on beta‐blocker (BB) therapy during the time of each Holter recording. Regular follow‐ups with Holter ECG recordings are recommended for children on prophylactic beta‐blocker therapy to assist in evaluating heart rate and treatment response. Each patient in the study had completed at least one digital Holter recording during their normal daily activities. All Holter ECG recordings clinically stored between 2000 and 2021 were retrieved.

The Holter recordings were performed with different digital recorders during the 20‐year observation period. They were analyzed with either the Aspect Holter system (GE Healthcare, Stockholm, Sweden) or Pathfinder SL software (Spacelabs Healthcare, Hertford, UK), where either lead V2 or lead V5 was analyzed. The ECG was sampled at 100 Hz, and R waves were detected using the built‐in software in the Holter system. Heart beats were classified as normal or abnormal due to arrhythmia during the Holter analysis. ECGs and annotations of beats were exported from the Holter systems.

### 2.3 Heart rate variability (HRV)

The analysis of HRV, that is, the beat‐to‐beat variation in the RR intervals, was performed using power spectrum analysis. RR intervals were adjusted after interpolating all ECGs to 1000 Hz sampling frequency using cubic spline interpolation. The RR interval data were converted to a time series by cubic spline interpolation, followed by re‐sampling at 5 Hz and removal of the mean value. Since our focus was on the assessment of cardiac autonomic function, we excluded all RR intervals that were classified as non‐sinus beats during the Holter analysis. Additionally, we used an algorithm to automatically remove misclassified premature beats and other aberrant heartbeats (Karlsson et al., 2012). If a RR interval differed more than 40% relative to the mean of the preceding and following RR intervals, it was considered abnormal and was removed. This threshold was selected as a tradeoff between removing RR intervals originating from extrasystolic beats and not removing variability due to excessive respiratory sinus arrhythmia. Data were divided into consecutive 5‐min segments; however, only 5‐min segments where >50% of RR intervals remained were included.

Power spectra were determined for all 5‐min segments based on autoregressive modeling with a fixed model order (30 parameters). This model order was selected to avoid underestimating the number of spectral peaks. The total power (PTOT) was calculated as the total area in the spectrum. The power of three spectral components was also calculated: the very low frequency component (VLF, the area in the spectrum below 0.04 Hz), the low‐frequency component (LF, the area in the region 0.04–0.15 Hz), and the high‐frequency component (HF, the area in the region 0.15–1.0 Hz). Thus, HRV indices from a maximum of 288 segments were determined in each recording. In addition, the LF/HF ratio and the mean heart rate (HR) were calculated. Regarding cardiac autonomic regulation, we considered HF to mainly represent parasympathetic activity and LF to reflect a combination of sympathetic and parasympathetic activity (Akselrod et al., 1981). All HRV analyses were performed using MATLAB ver. 2022b (MathWorks Inc., Natick, MA, USA).

### 2.4 Statistical analysis

All HRV variables were expressed in ms^2^ and log‐transformed with a base of 10. Two different analyses were performed based on linear mixed effects modeling.

Analysis 1: The differences between groups were analyzed by dividing the HRV indices from all 5‐min segments in each recording into 16 subgroups according to the mean HR in each segment and the subject's age at the recording: four HR regions (60–80 beats per minute (bpm), 80–100, 100–120, and 120–140 bpm) and four different age‐groups (1–5, 5–10, 10–15, and 15–18 years). In each HR range and age‐group, a mixed model analysis of HRV indices was performed with the group as a fixed effect and assuming a random intercept. Thus, we assumed that the eventual dependency of HR was small within each HR range.

Analysis 2: The specific short‐term effect of BB therapy was analyzed using patients for whom recordings were available from before and during BB therapy. In this analysis, the HRV indices from all 5‐min segments in each recording were divided according to the corresponding heart rate decade, and the average HR and HRV were calculated in each heart rate region. The overall relationship between HR and HRV indices was analyzed using linear mixed modeling, with group and HR as factors, and assuming a random intercept and slope at the subject level. In this analysis, HR was expressed as the deviation from 60 bpm; thus, the intercept corresponded to HRV at HR = 60 bpm and the slope represents the change in HRV when HR increases. This analysis was also used to analyze differences between female and male LQTS patients in different age‐groups.

The changes in HRV with increasing age, in different HR ranges, were estimated by a second‐order polynomial; this analysis was only performed for all recordings in LQT1 and controls, and 95% confidence intervals were also estimated for controls.

Since log‐transformed HRV data were used, a difference of −0.3 in logarithmic units scores corresponded to a 50% reduction in original values. Similarly, the difference −0.12 corresponded to a 25% reduction in absolute values.

All statistical tests were performed using significance *p* < .05. Analyses were performed using MATLAB R2022b (MathWorks Inc.).

## 3 RESULTS

### 3.1 Participants

**FIGURE 1:**
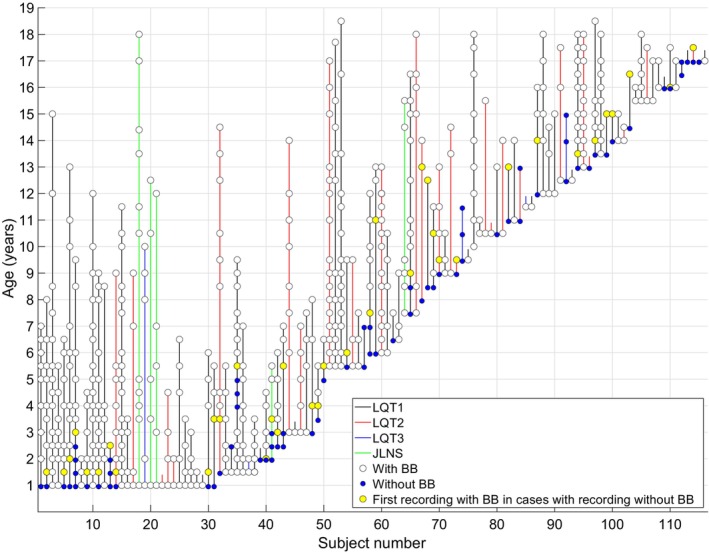
Overview of included Holter recordings per subject. Each vertical line represents one subject, and the circles mark the subject's age at each Holter recording. BB, beta‐blocker therapy; JLNS, Jervell and Lange‐Nielsen syndrome; LQT1, long QT syndrome type 1; LQT2, long QT syndrome type 2; LQT3, long QT syndrome type 3.

A total of 575 Holter recordings from the 116 LQTS patients were initially included. In the control group, each of the 69 healthy children and adolescents underwent a single Holter recording. Table 1 presents the subjects' clinical characteristics and the number of recordings. The majority of the LQTS patients had LQT1 (*n* = 80). The patients were followed longitudinally for a median of 3.5 years (year range 0–17, interquartile range, 1.0–6.0) (Figure 1). The number of subjects and recordings varied across different HRV analyses depending on the number of recordings in each age interval and after excluding Holter ECG segments with poor quality and excessive noise (Table S1). HRV was analyzed in a total of 176,837 segments, where 5.4% segments were excluded since less than 50% of RR intervals remained. In 90.4% of segments, more than 90% of detected RR intervals were used.

**TABLE 1:** Clinical characteristics of study participants.

	Controls	LQT1	LQT2	LQT3	JLNS
*N*	69	80	26	5	5
Female (%)	34 (49%)	44 (55%)	10 (38%)	0 (0%)	3 (60%)
Age range (years)	1–18	1–18	1–18	1–15	1–18
Recordings	69	401	126	20	37
Recordings per subject	1.0	5.0	4.7	3.3	7.4
Symptomatic (%)	0 (0%)	16 (20%)	8 (31%)	2 (40%)	3 (60%)
ICD/PM	0	2	1	2	4

Abbreviations: ICD/PM, intracardiac defibrillator/pacemaker; JLNS, Jervell and Lange‐Nielsen syndrome; LQT1, long QT syndrome type 1; LQT2, long QT syndrome type 2; LQT3, long QT syndrome type 3; *N* = number of subjects.

### 3.2 HRV in different LQTS genotypes

**FIGURE 2:**
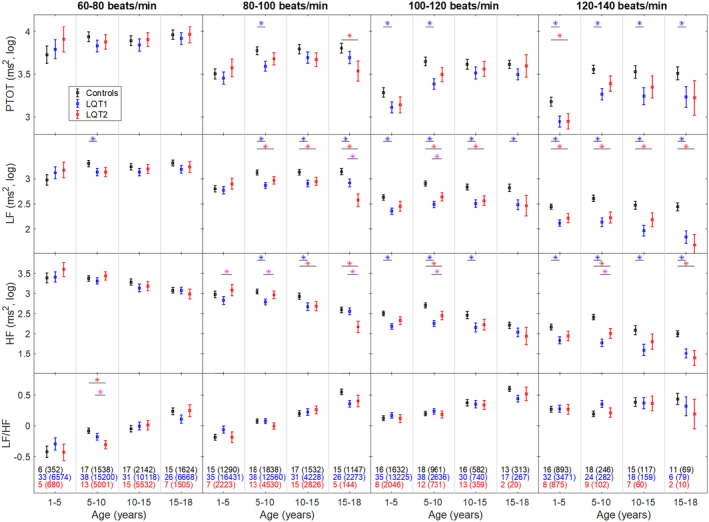
Heart rate variability in LQT1, LQT2, and controls. Boxplots show mean ± SE, calculated with a mixed model analysis after dividing data from all 5‐min segments in 16 subgroups based on heart rate and age. Values are log‐10 transformed HRV expressed in ms^2^. HF, power of the high‐frequency component; LF, power of the low‐frequency component; LF/HF, the ratio between the low‐frequency component and the high‐frequency component; LQT1, long QT syndrome type 1; LQT2, long QT syndrome type 2; PTOT, total power. Blue * indicates significant differences between LQT1 and controls. Red * indicates significant differences between LQT2 and controls. Purple * indicates significant differences between LQT1 and LQT2. Black, blue, and red numbers show the number of subjects in each age‐group, and the number in parentheses shows the number of analyzed 5‐min segments.

When dividing the LQTS patients into subgroups according to genotype and age, significant differences in HRV indices could be seen between both LQT1 and LQT2 patients compared with controls (Figure 2, Table S1). These findings emerge when the HR increases, where both LF and HF were significantly lower in many of the LQT1 and LQT2 subgroups compared with controls. However, some of the LQT2 subgroups were small, especially in the age 15–18 years (Table S1). In all LQT1 age‐groups, at a HR between 120 and 140 bpm, the PTOT was significantly lower compared with controls (Table S1).

When comparing LQT1 and LQT2 (Figure 2), no significant differences in PTOT were found. However, regarding HF in the HR interval 80–100 bpm, LQT1 patients between 1 and 10 years of age had a lower HF than LQT2 patients (1–5 years: *p* = .05; 5–10 years: *p* = .02). In the same HR interval, LQT2 patients between 15 and 18 years of age had a lower HF than LQT1 patients (*p* < .01). In the HR interval 100–140 bpm, LQT1 patients between 5 and 10 years of age had a lower HF than LQT2 patients (HR 100–120: *p* = .03; HR 120–140: *p* = .04). Regarding LF in the HR interval 80–100 bpm, LQT2 patients between 15 and 18 years of age had a lower LF than LQT1 patients (*p* < .01). In the HR interval 100–120 bpm, LQT1 patients between 5 and 10 years of age had a lower LF than LQT2 patients (*p* = .05).

HRV parameters in LQT3 and JLNS are shown in Table S1. However, conclusions are hard to make since the LQT3 and JLNS groups were small. Nonetheless, a tendency was seen for these subjects also presented with a lower PTOT, LF, and HF compared with controls.

### 3.3 Symptomatic LQT1 and LQT2

**FIGURE 3:**
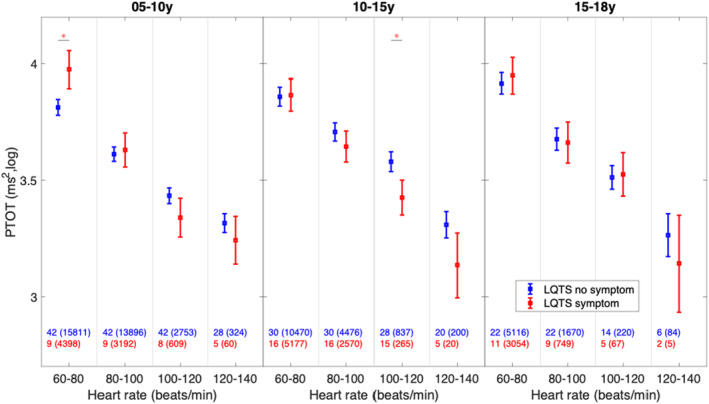
Heart rate variability in asymptomatic and symptomatic patients with long QT syndrome (LQTS). Boxplots show mean ± SE, calculated with a mixed model analysis in the different age and heart rate groups. PTOT, total power. * indicates significant differences between asymptomatic and symptomatic LQTS patients. Blue and red numbers show the number of subjects in each heart rate region, and the number in parentheses shows the number of analyzed 5‐min segments.

Sixteen LQT1 patients (20%) and eight LQT2 patients (31%) had been clinically classified as symptomatic. All were on BB therapy. These 24 symptomatic patients were compared with the remaining 64 asymptomatic LQT1 and 18 LQT2 patients (*n* = 82). A significantly lower PTOT could be seen in symptomatic patients 10–15 years at a HR between 100 and 120 bpm (*p* = .04). At a HR between 60 and 80 bpm, a lower PTOT could be noticed in asymptomatic patients at the age‐group 5–10 years (*p* = .05) (Figure 3 and Table S3). However, it should be worth noted that these subgroups were small.

### 3.4 Sex‐ and age‐related HRV

When considering differences between the two sexes in the LQT1 patients, females between the ages 10 and 18 years had a significantly lower PTOT (10–15 years: *p* = .04; 15–18 years: *p* = .02) than males (Table S3).

Figure 2 shows that HRV parameters in both LQTS patients and controls decrease as HR increases. Additionally, an age‐dependent pattern in HRV was observed, and this was estimated by a quadratic age‐related model (Figure S1). In controls, PTOT, LF, and HF increase from early age and to a maximum value, although the age where the peak occurs varies between HRV indices. For LQT1 patients, the age dependency in PTOT peaked at 10 years of age in all HR regions, but PTOT subsequently deviates progressively as both age and HR increase. LQT1 patients also presented with nearly linear age dependency in LF and especially in HF, with no increase from 1 year of age noted in three quarters of the HR regions.

### 3.5 HRV in LQTS patients before and after start of beta‐blocker therapy

**FIGURE 4:**
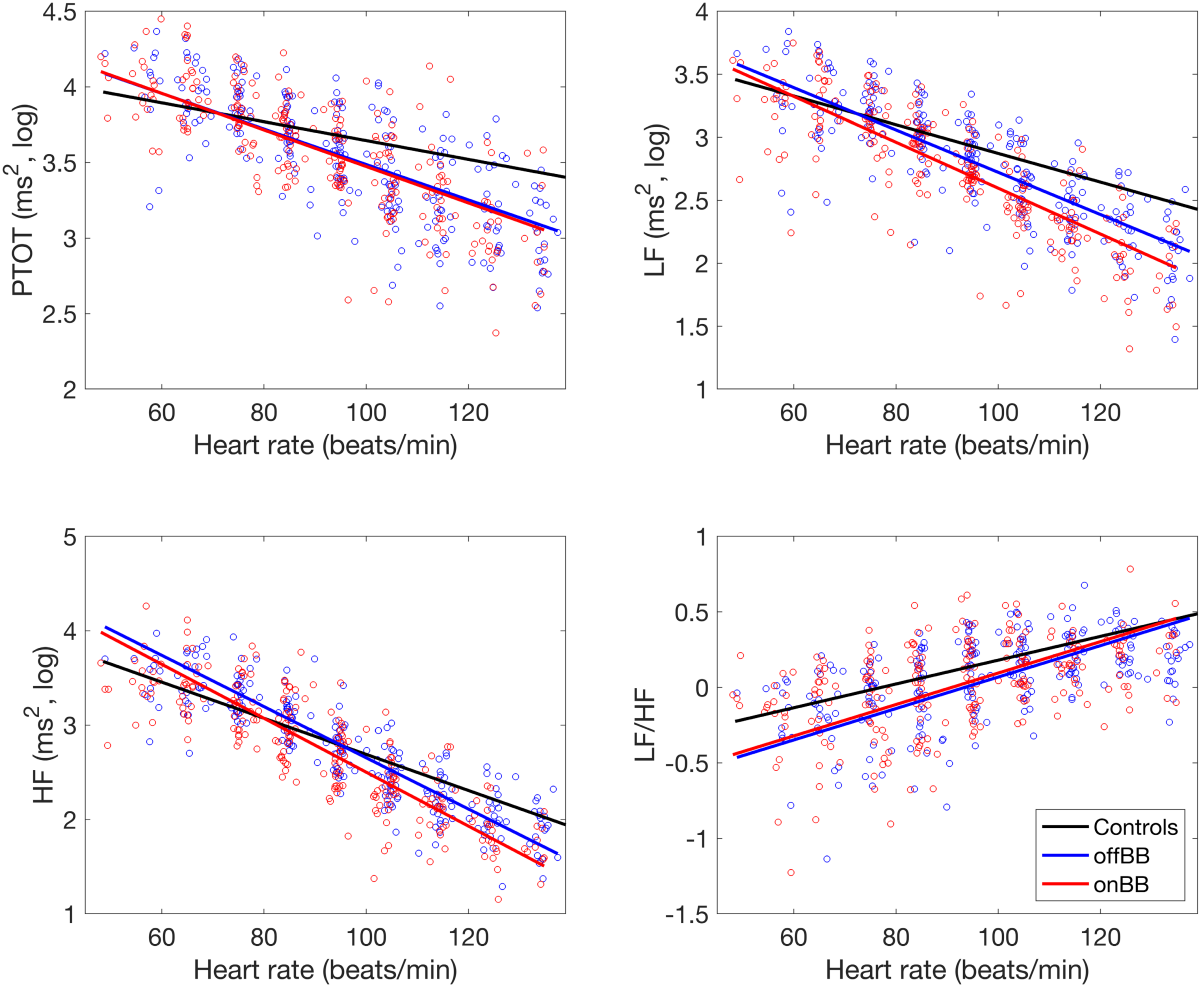
Relationship between heart rate and heart rate variability before and after initiation of beta‐blocker therapy in LQTS patients. Points show the average HR and HRV in each heart rate decade for each recording in patients. PTOT, total power; HF, power of the high‐frequency component; LF, power of the low‐frequency component; LF/HF, the ratio between the low‐frequency component and the high‐frequency component; offBB, long QT patients off beta‐blocker therapy; onBB, long QT patients on beta‐blocker therapy.

Of the 116 included LQTS patients, 37 had a Holter recording both before and after the start of BB therapy. Eighteen were female (49%), 30 had genetically verified LQT1, six had LQT2, and one had JLNS. Five were classified as symptomatic (LQT1: *n* = 3, LQT2: *n* = 1, JLNS: *n* = 1). The mean age without BB was 6.4 ± 5.1 years, and on BB therapy, it was 7.6 ± 5.4 years. After initiation of BB therapy, 28 received propranolol and nine metoprolol. Compared to the control group, LQTS patients without BB therapy showed a significantly steeper slope in PTOT (*p* < .001), LF (*p* < .001), and HF (*p* < .001) curves, leading to more pronounced differences in higher HR ranges. This is observed in Figure 4 and Table S4. In a pairwise comparison of Holter recordings in LQTS patients before and after initiating BB therapy, significant differences were observed in the slopes of both the LF (*p* < .001) and HF (*p* < .001) components. It is important to emphasize that despite statistical significance, the actual difference in effect size was minimal. Furthermore, patients not receiving BB therapy had a significantly higher intercept for both the LF (*p* < .001) and HF (*p* < .001) components when compared to those on BB therapy. However, this observation is likely attributable to a leftward shift of the curve caused by the decreased HR due to BB therapy.

## 4 DISCUSSION

The objective of this study was to conduct a retrospective assessment of HRV in Holter recordings among children and adolescents diagnosed with long QT syndrome, with a focus on cardiac autonomic activity. The key finding in this study was the link between HR and changes in HRV parameters. When HR was low, no differences in HRV were found between LQTS patients and controls, nor between different genotypes or between sexes. However, with increasing HR, differences in HRV emerged and overall, the HRV parameters tended to be lower in the LQTS group.

Holter recordings are routinely conducted in this specific group of patients to identify dynamic changes like T‐wave alternans or Torsades de Pointes (TdP), as well as to monitor medication adherence (Mauriello et al., 2011). However, there is a scarcity of studies investigating HRV in children with LQTS, and to the best of our knowledge, none have previously examined HRV in 24‐h Holter recordings within a selected pediatric and adolescent population. Considering that HRV is regarded as a reflection of the autonomic influence on the heart, it can be hypothesized that HRV abnormalities observed in LQTS patients may be valuable when studying the role of the cardiac ANS in the pathogenesis of LQTS.

Previous studies on HRV in LQTS patients have been based on a small number of subjects, and the results have been somewhat contradictory. In one study involving 13 LQTS patients off BB, no differences in LF and HF power were observed compared to healthy controls (Morita et al., 1996). Another study found increased HF and decreased LF in 12 adult LQTS patients who were off BB (Shamsuzzaman, 2003). However, one study focusing on 27 adult LQTS mixed genotype carriers and their non‐carrier family members observed no differences in the HRV parameters, and the analysis was performed in both medicated and non‐medicated patients (Perkiömäki et al., 2001). More recently, DeMaria et al. (2020) reported that adult LQTS patients had reduced vagal tone and total variability compared to controls on Holter recordings. It is important to highlight that these studies mainly included adult subjects, and factors such as genotype, age dependency, or HR were often not considered. In our study of a pediatric and adolescent LQTS cohort, we could show that the HRV parameters are HR‐dependent which may explain the divergent results in previous studies.

### 4.1 HRV depending on genotypes

In the comparison between LQT1 and LQT2, LQT1 patients showed a lower HF than LQT2 patients in the two youngest age‐groups. A lower HF indicates low parasympathetic activity, which in turn may contribute to a disturbed autonomic balance with sympathetic dominance and risk of cardiac events. Several clinical studies have demonstrated an age‐ and genotype‐dependent difference in risk in LQT1 and LQT2 (Kutyifa et al., 2018; Locati et al., 1998; Zareba et al., 1998). Our HRV findings in children with LQT1 may correlate to the higher risk for cardiac events in the lower age‐group compared to LQT2.

With increasing age, the HRV parameters shifted between genotypes, with the LQT2 patients showing a lower HF and LF than the LQT1 patients with increasing age. This was significant in the HR interval 80–100 bpm, but the same tendency could be seen in the other HR intervals. This observation is consistent with Perkiömäki et al.'s study, which showed that, although not statistically significant, adult LQT2 patients tended to have lower HRV values than LQT1 patients (Perkiömäki et al., 2001). Once again, our age‐ and genotype‐related results suggest that low HRV parameters are consistent with the clinical findings in previous studies regarding the risk of cardiac events in LQT1 and LQT2 (Kutyifa et al., 2018; Younis et al., 2023; Zareba et al., 1998).

### 4.2 HRV in symptomatic LQTS patients

The few studies conducted in LQTS patients on autonomic control and risk of cardiac events show divergent results. Schwartz et al. (2008) found that baroreflex sensitivity was higher in symptomatic LQT1, whereas HRV did not seem to distinguish between symptomatic and asymptomatic individuals. In a Holter study by Porta et al. (2015), the main finding was greater reactivity of the vagal control of the HR in symptomatic mutation carriers. It should be noted, however, that in both studies, LQT1 patients from a KCNQ1 founder population were examined. A founder population creates a genetic subset diverging from that of the general LQT1 population, and these patients may have different genetic modifiers that may affect the autonomic response. In contrast, DeMaria et al. (2020) found that in a LQT1 cohort consisting of different genetic variants, the individuals suffering from arrhythmias showed a lower PTOT and vagal function on Holter recordings. These results were found across multiple physiological states and are in agreement with our study. Notably, none of the aforementioned studies examined younger individuals, which makes our results even more interesting. We observed a significantly lower PTOT among symptomatic individuals aged 10–15 years at a HR between 100 and 120 bpm. Because a lower PTOT indicates a higher risk of cardiac events, our findings are consistent with the well‐established fact that the probability of experiencing a first event before the age of 15 is higher. It is also widely recognized that the risk in this age‐group is higher for males. In our symptomatic subgroup of LQTS patients aged 10–15 years, at a HR between 100 and 120 bpm, eight out of 15 patients with symptoms were male.

### 4.3 HRV depending on sex and age

Prepubertal males and postpubertal females with LQTS have a higher risk of arrhythmic events, underscoring the importance of studying a pediatric and adolescent population (Hobbs et al., 2006; Locati et al., 1998; Zareba et al., 2003). In this study, we found that females with LQTS aged 10–18 years had a significantly lower PTOT than males of the same age. This difference in HRV between sexes during adolescence is noteworthy, particularly considering the recognized age‐ and sex‐related variations in the risk of cardiac events. A reduction in total spectral power is linked to an increased risk of ventricular arrhythmias, as demonstrated in numerous studies (Battipaglia et al., 2010, 2012; Galinier, 2000; La Rovere et al., 2001; Malik et al., 1996). One would have expected lower PTOT values in LQTS males in the 10–15 years of age‐group. However, the age‐groups are small, and the gender distribution is not equal, especially in the 10–15 years of age‐group, which may affect the findings. Nonetheless, in the postpubertal age‐groups (15–18 years), there is a clear difference in PTOT, with females showing significantly lower power compared to males. This observation may indicate a higher risk of cardiac events.

When examining age development in HRV components, LQTS patients and controls followed the same pattern but on different levels. LQTS patients generally had lower values, with increasing deviation at increasing HR. This may indicate a lower cardiac autonomic response during and after HR‐increasing activities. The results align with a prior study conducted by our research team, where adult patients with LQTS displayed lower LF and LF/HF ratios during peak exercise, and lower PTOT, LF, and HF during the recovery phase compared to the control group Lundström et al., 2021. These findings suggest that LQTS patients demonstrate a different pattern of autonomic reactivation following exercise, characterized by diminished parasympathetic activity. Collectively, these observations could imply that LQTS patients have a deviated autonomic response during activities that increase HR, potentially resulting in an imbalance within the ANS.

### 4.4 LQTS patient on and off beta‐blocker therapy

In this study, we found that with increasing HR, the LQTS patients off BB significantly decreased in PTOT, LF, and HF compared with controls. As mentioned previously, reduced HRV components, and especially a decreased PTOT, are linked to an increased risk of ventricular arrhythmias in various other cardiac conditions (Battipaglia et al., 2010, 2012; Galinier, 2000; La Rovere et al., 2001; Malik et al., 1996). This makes the lower HRV power at higher HR in LQTS patients off BB compared to controls even more interesting. Thus, it would appear logical to assume that those patients with LQTS who present with reduced HRV might have a higher risk for ventricular arrhythmias than those with normal HRV findings.

The pair‐wise comparison of LQTS patients off and on BB therapy showed differences in the LF and HF components. However, the actual difference in effect size in the slope was negligible, and the difference in the intercept probably is due to a left shift of the curve, induces by the natural decrease in HR with BB therapy. Therefore, in this study population, BB therapy had a minimal impact on HRV components. This aligns with Hayano et al. (1991) findings, who observed no immediate effect on any HRV components following propranolol injection. Other studies on BB therapy effects on HRV have reported a decrease in the LF component (Pagani et al., 1986; Pomeranz et al., 1985). However, there is variability in the findings regarding the HF component, including reports of increase (Pagani et al., 1986; Vaile et al., 1999; Chen et al., 2012), and no effect (Pomeranz et al., 1985) with BB therapy. Altogether, this makes it difficult to draw conclusions regarding BB therapies' actual effect on HRV, and a more extensive study is needed if this should be clarified.

### 4.5 The advantage of not correcting HRV for heart rate

This study undertakes a novel approach by analyzing HRV across different HR ranges and age‐groups. HRV is known to depend on mean HR and increases from birth until adolescence and then successively decreases. Recent studies suggest that HRV should be adjusted for HR, but methods vary (Monfredi et al., 2014; Sacha et al., 2013; van Roon et al., 2016). Furthermore, different age‐dependency corrections have also been suggested, including age‐adjusting based on estimated linear or polynomial regression lines and, in some cases, also converting data to z‐scores. Our decision not to adjust for HR proved advantageous, as it allowed us to detect HRV differences at higher HRs. This approach has the advantage of not requiring linear or non‐linear models, but it may be limited to recordings with large HR variation, like those spanning over 24 h.

### 4.6 Study protocol

The participants did not follow a standardized protocol during the 24‐h Holter recordings. Some LQTS children may have had restrictions regarding the activity level. However, Gow et al. (2013) demonstrated that the activity levels often exceeded prior recommendations. Therefore, in 2015, the ECS guidelines removed previous activity restrictions for LQTS children (Priori et al., 2015). Moreover, the control subjects did not perform any strenuous activity during the recording, and our analysis also included the nighttime period. Therefore, patients and controls were investigated during relatively similar conditions, as recommended in the Task Force report (Malik et al., 1996). In this study, we did not perform separate analyses of data from the daytime and nighttime periods. However, the most important findings were observed during periods with high heart rates which mainly occur during daytime.

### 4.7 Limitations of the study

An unequal number of repeated measurements across subjects and a small number of LQT2, and particularly LQT3 and JLNS patients, prevented a comprehensive analysis of genotype‐based differences due to small sample sizes.

The study's retrospective nature determined the number of patients on and off BB therapy, and ethical considerations prevented BB discontinuation for additional recordings. Two BB types were administered, historically considered equally effective, but recent studies show that propranolol appears to outperform metoprolol in shortening QTc interval and preventing cardiac events (Chatrath et al., 2004; Chockalingam et al., 2012). Most participants were prescribed propranolol. Nadolol was not used due to unavailability in Sweden.

## 5 CONCLUSIONS

A key finding in this study was the correlation between HR and changes in HRV parameters, where LQTS patients showed an atypical autonomic response with increasing HR compared with controls. Interestingly, the findings of deviated HRV components regarding genotype, age, and sex in LQTS patients also correspond to the clinically well‐known phenotype in LQTS. This study highlights a possible link between abnormal autonomic response and risk for arrhythmias in LQTS.

## AUTHOR CONTRIBUTIONS

A.L., A.R. and U.W., all contributed to the conception of the work. Data collection were performed by A.L and M.K. with contribution from H.E. U.W. designed and performed the analysis. The initial draft of the document was written by A.L. The manuscript was revised and approved by A.R. and U.W. The article's submission was reviewed and approved by all authors.

## FUNDING INFORMATION

This work was supported by the Swedish Heart Lung Foundation and through the Regional Agreement between Umeå University and Region Västerbotten (ALF).

## CONFLICT OF INTEREST STATEMENT

The authors have no conflicts of interest to disclose.

## ETHICS STATEMENT

The study was conducted according to the principles of the Declaration of Helsinki. Informed consent was obtained from the legal guardians. The Regional Ethical Review Board in Umeå, Sweden, approved the study (Dnr: 05‐127M).

## Supporting information


Data S1:

## Data Availability

The data that support the findings of this study are available on request from the corresponding author. The data are not publicly available due to privacy or ethical restrictions.
