# Supplementary material for: Holter study of heart rate variability in children and adolescents with long QT syndrome
Source: Ann Noninvasive Electrocardiol. 2024 Jun 18;29(4):e13132. doi: 10.1111/anec.13132 (PMC11184570; doi:10.1111/anec.13132)
Supplement: Supplementary file 1 — Data S1: [file ANEC-29-e13132-s001.docx]

# Supplementary to the article: Holter Study of Heart Rate Variability in Children and Adolescents with Long QT Syndrome

Anna Lundström^a¶^, Håkan Eliasson^c^, Marcus Karlsson^b^, Urban Wiklund^b&^ and Annika Rydberg^1&^

^a^ Department of Clinical Sciences, Umeå University, Umeå, Sweden,

^b^ Department of Diagnostics and Intervention, Radiation Physics, Biomedical Engineering, Umeå University, Umeå, Sweden

^c^ Department of Women´s and Children´s Health, Karolinska Institutet, Stockholm, Sweden

^&^These authors also contributed equally to this work.

| **Table S1.** Heart rate variability in patients with Long QT syndrome type 1 (LQT1), type 2 (LQT2), type 3 (LQT3), Jervell and Lange-Nielsen syndrome (JLNS) and controls. | | | | | | | |  |
| --- | --- | --- | --- | --- | --- | --- | --- | --- |
| Group | HR (beats/min) | Age (years) | Nsubj (Nseg) | PTOT  (ms^2^, log) | LF  (ms^2^, log) | HF  (ms^2^, log) | LF/HF | |
| Controls | 60-80 | 1-5 | 6(353) | 3.73(0.10) | 2.98(0.11) | 3.39(0.12) | -0.42(0.09) | |
|  |  | 5-10 | 17(1537) | 3.94(0.06) | 3.30(0.06) | 3.38(0.07) | -0.08(0.05) | |
|  |  | 10-15 | 17(2143) | 3.89(0.06) | 3.24(0.06) | 3.29(0.08) | -0.05(0.05) | |
|  |  | 15-18 | 15(1624) | 3.96(0.05) | 3.32(0.06) | 3.08(0.07) | 0.24(0.05) | |
|  | 80-100 | 1-5 | 15(1289) | 3.50(0.06) | 2.80(0.06) | 2.98(0.08) | -0.18(0.05) | |
|  |  | 5-10 | 18(1839) | 3.77(0.05) | 3.12(0.05) | 3.05(0.06) | 0.08(0.03) | |
|  |  | 10-15 | 17(1534) | 3.79(0.05) | 3.13(0.05) | 2.93(0.07) | 0.20(0.04) | |
|  |  | 15-18 | 15(1148) | 3.80(0.06) | 3.15(0.06) | 2.59(0.07) | 0.55(0.04) | |
|  | 100-120 | 1-5 | 16(1632) | 3.29(0.05) | 2.63(0.06) | 2.51(0.06) | 0.12(0.04) | |
|  |  | 5-10 | 18(961) | 3.65(0.05) | 2.91(0.05) | 2.71(0.06) | 0.20(0.04) | |
|  |  | 10-15 | 16(583) | 3.61(0.06) | 2.84(0.06) | 2.46(0.09) | 0.37(0.05) | |
|  |  | 15-18 | 13(314) | 3.62(0.05) | 2.82(0.07) | 2.21(0.08) | 0.60(0.04) | |
|  | 120-140 | 1-5 | 16(896) | 3.18(0.05) | 2.44(0.05) | 2.17(0.07) | 0.27(0.05) | |
|  |  | 5-10 | 18(246) | 3.56(0.05) | 2.61(0.06) | 2.41(0.07) | 0.19(0.04) | |
|  |  | 10-15 | 15(118) | 3.53(0.07) | 2.47(0.08) | 2.09(0.11) | 0.39(0.07) | |
|  |  | 15-18 | 11(68) | 3.51(0.07) | 2.44(0.08) | 2.00(0.07) | 0.44(0.09) | |
| LQT1 | 60-80 | 1-5 | 33(6574) | 3.79(0.11) | 3.12(0.12) | 3.41(0.13) | -0.30(0.10) | |
|  |  | 5-10 | 38(15200) | 3.83(0.07) | 3.14(0.07)* | 3.32(0.08) | -0.18(0.05) | |
|  |  | 10-15 | 31(10118) | 3.84(0.07) | 3.14(0.08) | 3.14(0.10) | -0.00(0.06) | |
|  |  | 15-18 | 26(6668) | 3.92(0.07) | 3.19(0.07) | 3.08(0.09) | 0.11(0.07) | |
|  | 80-100 | 1-5 | 35(16431) | 3.45(0.07) | 2.77(0.07) | 2.83(0.10) | -0.06(0.06)* | |
|  |  | 5-10 | 38(12560) | 3.59(0.06)* | 2.87(0.06)* | 2.79(0.07)* | 0.08(0.04) | |
|  |  | 10-15 | 31(4228) | 3.69(0.07) | 2.91(0.07)* | 2.68(0.09)* | 0.23(0.06) | |
|  |  | 15-18 | 26(2273) | 3.69(0.07) | 2.92(0.08)* | 2.56(0.08) | 0.36(0.06)* | |
|  | 100-120 | 1-5 | 35(13225) | 3.11(0.07)* | 2.35(0.07)* | 2.18(0.07)* | 0.17(0.05) | |
|  |  | 5-10 | 38(2636) | 3.39(0.06)* | 2.49(0.06)* | 2.25(0.08)* | 0.23(0.04) | |
|  |  | 10-15 | 30(740) | 3.51(0.07) | 2.51(0.08)* | 2.16(0.11)* | 0.35(0.06) | |
|  |  | 15-18 | 17(267) | 3.50(0.06) | 2.48(0.10)* | 2.04(0.11) | 0.44(0.05)* | |
|  | 120-140 | 1-5 | 32(3471) | 2.95(0.06)* | 2.11(0.06)* | 1.84(0.09)* | 0.28(0.06) | |
|  |  | 5-10 | 24(282) | 3.27(0.07)* | 2.13(0.08)* | 1.78(0.09)* | 0.36(0.06)* | |
|  |  | 10-15 | 18(159) | 3.24(0.10)* | 1.96(0.11)* | 1.59(0.15)* | 0.37(0.09) | |
|  |  | 15-18 | 6(79) | 3.24(0.12)* | 1.84(0.13)* | 1.51(0.12)* | 0.32(0.15) | |
| LQT2 | 60-80 | 1-5 | 5 (680) | 3.91(0.15) | 3.18(0.16) | 3.60(0.18) | -0.43(0.13) | |
|  |  | 5-10 | 13 (5001) | 3.88(0.09) | 3.13(0.09) | 3.44(0.10) | -0.31(0.07)* | |
|  |  | 10-15 | 15 (5532) | 3.90(0.08) | 3.20(0.09) | 3.19(0.11) | 0.01(0.08) | |
|  |  | 15-18 | 7 (1505) | 3.96(0.10) | 3.24(0.10) | 2.99(0.12) | 0.25(0.09) | |
|  | 80-100 | 1-5 | 7 (2225) | 3.57(0.10) | 2.90(0.11) | 3.09(0.14) | -0.19(0.09) | |
|  |  | 5-10 | 13 (4530) | 3.68(0.07) | 2.97(0.08)* | 2.97(0.09) | -0.00(0.05) | |
|  |  | 10-15 | 15 (2826) | 3.67(0.08) | 2.95(0.08)* | 2.68(0.11)* | 0.26(0.07) | |
|  |  | 15-18 | 5 (144) | 3.54(0.12)* | 2.57(0.13)* | 2.17(0.13)* | 0.40(0.09) | |
|  | 100-120 | 1-5 | 8 (2046) | 3.14(0.09) | 2.45(0.10) | 2.33(0.10) | 0.12(0.06) | |
|  |  | 5-10 | 12 (731) | 3.50(0.08) | 2.64(0.08)* | 2.45(0.10)* | 0.19(0.06) | |
|  |  | 10-15 | 13 (359) | 3.56(0.09) | 2.56(0.09)* | 2.22(0.13) | 0.34(0.07) | |
|  |  | 15-18 | 2 (20) | 3.60(0.13) | 2.46(0.20) | 1.95(0.22) | 0.52(0.11) | |
|  | 120-140 | 1-5 | 8 (875) | 2.95(0.09)* | 2.22(0.09)* | 1.95(0.12) | 0.27(0.08) | |
|  |  | 5-10 | 9 (102) | 3.39(0.09) | 2.22(0.11)* | 2.01(0.12)* | 0.21(0.08) | |
|  |  | 10-15 | 7 (60) | 3.35(0.13) | 2.18(0.14)* | 1.81(0.19) | 0.36(0.12) | |
|  |  | 15-18 | 2 (10) | 3.22(0.20) | 1.67(0.21)* | 1.39(0.19)* | 0.19(0.24) | |
| LQT3 | 60-80 | 1-5 | 1 (162) | 4.05(0.26) | 3.42(0.28) | 3.64(0.31) | -0.22(0.23) | |
|  |  | 5-10 | 2 (462) | 3.37(0.17)* | 2.73(0.18)* | 2.35(0.20)* | 0.38(0.13)* | |
|  |  | 10-15 | 4 (627) | 3.54(0.13)* | 2.89(0.14)* | 2.49(0.18)* | 0.40(0.12)* | |
|  |  | 15-18 | 1 (114) | 3.55(0.22) | 2.94(0.24) | 2.76(0.27) | 0.19(0.21) | |
|  | 80-100 | 1-5 | 2 (470) | 3.40(0.17) | 2.71(0.18) | 2.68(0.23) | 0.04(0.14) | |
|  |  | 5-10 | 2 (216) | 3.45(0.15)* | 2.77(0.16)* | 2.36(0.19)* | 0.41(0.11)* | |
|  |  | 10-15 | 4 (445) | 3.56(0.12) | 2.77(0.12)* | 2.37(0.17)* | 0.40(0.10) | |
|  |  | 15-18 | 1 (102) | 3.27(0.22)* | 2.77(0.24) | 2.20(0.26) | 0.57(0.18) | |
|  | 100-120 | 1-5 | 2 (447) | 3.16(0.16) | 2.47(0.16) | 2.19(0.17) | 0.28(0.11) | |
|  |  | 5-10 | 2 (63) | 3.40(0.16) | 2.44(0.17)* | 2.01(0.20)* | 0.43(0.12)* | |
|  |  | 10-15 | 3 (320) | 3.43(0.14) | 2.69(0.15) | 2.30(0.22) | 0.39(0.12) | |
|  |  | 15-18 | 1 (59) | 3.31(0.15) | 2.54(0.26) | 1.84(0.29) | 0.71(0.14) | |
|  | 120-140 | 1-5 | 1 (53) | 2.88(0.21) | 2.23(0.21) | 1.88(0.29) | 0.35(0.20) | |
|  |  | 5-10 | 2 (7) | 3.31(0.17) | 1.94(0.21)* | 1.46(0.23)* | 0.48(0.14)* | |
|  |  | 10-15 | 3 (46) | 3.29(0.18) | 2.42(0.19) | 1.83(0.27) | 0.62(0.16) | |
|  |  | 15-18 | 1 (11) | 3.02(0.23)* | 2.09(0.24) | 1.15(0.22)* | 0.94(0.30) | |
| JLNS | 60-80 | 1-5 | 4(1073) | 3.65(0.16) | 2.71(0.17) | 3.26(0.19) | -0.55(0.14) | |
|  |  | 5-10 | 5(2299) | 3.61(0.12)* | 2.66(0.13)* | 3.10(0.14)* | -0.44(0.09)* | |
|  |  | 10-15 | 4(1662) | 3.54(0.13)* | 2.57(0.14)* | 2.75(0.18)* | -0.18(0.12) | |
|  |  | 15-18 | 2(461) | 3.58(0.16)* | 2.63(0.17)* | 2.70(0.20) | -0.07(0.15)* | |
|  | 80-100 | 1-5 | 4(1556) | 3.13(0.13)* | 2.22(0.13)* | 2.62(0.17)* | -0.40(0.10)* | |
|  |  | 5-10 | 5(593) | 3.29(0.10)* | 2.26(0.11)* | 2.60(0.13)* | -0.34(0.07)* | |
|  |  | 10-15 | 4(394) | 3.38(0.13)* | 2.21(0.13)* | 2.25(0.17)* | -0.05(0.10)* | |
|  |  | 15-18 | 1(28) | 3.20(0.23)* | 2.28(0.25)* | 2.36(0.26) | -0.08(0.18)* | |
|  | 100-120 | 1-5 | 4(441) | 2.82(0.12)* | 2.15(0.12)* | 2.20(0.12)* | -0.05(0.08)* | |
|  |  | 5-10 |  |  |  |  |  | |
|  |  | 10-15 |  |  |  |  |  | |
|  |  | 15-18 |  |  |  |  |  | |
|  | 120-140 | 1-5 | 2(117) | 3.43(0.16) | 2.77(0.16)* | 2.61(0.21)* | 0.16(0.14) | |
|  |  | 5-10 | 0 (0) |  |  |  |  | |
|  |  | 10-15 | 0 (0) |  |  |  |  | |
|  |  | 15-18 | 0 (0) |  |  |  |  | |

HR and HRV based on data from all 5-min segments in each 24-hour Holter recording, indices analyzed in different age groups and heart rate regions based on linear effects mixed modelling. HRV data are presented as estimated marginal means (SE). Data divided into different age groups (1-5 years, 5-10 years, 10-15 years and 15-18 years), heart rate (HR) regions (60-80 beats per minute (bpm), 80-100 bpm, 100-120bpm, 120-140bpm) and genotypes (LQT1, LQT2, LQT3, JLNS). Age presented in years. HR = heart rate; Nsubj = the number of subjects in each age group; Nseg = the number of 5 minutes Holter segments; PTOT = total power; LF = power of the low frequency component; HF = power of the high frequency component; LF/HF = the ratio between the low frequency component and the high frequency component; LQT1 = Long QT syndrome type 1; LQT2 = Long QT syndrome type 2; LQT3 = Long QT syndrome type 3; JLNS = Jervell and Lange-Nielsen syndrome. * indicate significant differences compared to controls (p<0.05).

**Table S2.** Heart rate variability in asymptomatic and symptomatic patients with Long QT syndrome (LQTS).

| Group | Age (years) | HR (beats/min) | Nsubj (Nseg) | PTOT  (ms^2^, log) | LF (ms^2^, log) | HF  (ms^2^, log) | LF/HF |
| --- | --- | --- | --- | --- | --- | --- | --- |
| Asymptomatic LQTS | 5-10 | 60-80 | 42(15803) | 3.81(0.03) | 3.11(0.04) | 3.32(0.04) | -0.21(0.03) |
|  |  | 80-100 | 42(13901) | 3.61(0.03) | 2.89(0.03) | 2.84(0.04) | 0.05(0.02) |
|  |  | 100-120 | 42(2754) | 3.43(0.03) | 2.52(0.03) | 2.30(0.04) | 0.23(0.02) |
|  |  | 120-140 | 28(324) | 3.31(0.04) | 2.17(0.05) | 1.86(0.06) | 0.31(0.04) |
|  | 10-15 | 60-80 | 30(10474) | 3.86(0.04) | 3.16(0.05) | 3.20(0.05) | -0.04(0.04) |
|  |  | 80-100 | 30(4477) | 3.71(0.04) | 2.95(0.04) | 2.74(0.05) | 0.21(0.03) |
|  |  | 100-120 | 28(837) | 3.58(0.04) | 2.54(0.05) | 2.19(0.06) | 0.35(0.03) |
|  |  | 120-140 | 20(199) | 3.30(0.06) | 2.07(0.07) | 1.68(0.09) | 0.38(0.05) |
|  | 15-18 | 60-80 | 22(5476) | 3.91(0.05) | 3.17(0.05) | 3.07(0.06) | 0.10(0.05) |
|  |  | 80-100 | 22(1867) | 3.66(0.05) | 2.90(0.06) | 2.53(0.06) | 0.37(0.04) |
|  |  | 100-120 | 16(311) | 3.45(0.05) | 2.44(0.08) | 1.96(0.08) | 0.49(0.03) |
|  |  | 120-140 | 8(89) | 3.24(0.08) | 1.85(0.11) | 1.55(0.11) | 0.28(0.10) |
| Symptomatic LQTS | 5-10 | 60-80 | 9(4398) | 3.97(0.08)* | 3.27(0.09)* | 3.47(0.10) | -0.20(0.07) |
|  |  | 80-100 | 9(3189) | 3.63(0.07) | 2.93(0.08) | 2.83(0.09) | 0.09(0.05) |
|  |  | 100-120 | 8(613) | 3.32(0.09) | 2.52(0.09) | 2.32(0.10) | 0.20(0.06) |
|  |  | 120-140 | 5(60) | 3.23(0.10) | 2.07(0.13) | 1.71(0.15) | 0.36(0.09) |
|  | 10-15 | 60-80 | 16(5176) | 3.86(0.07) | 3.15(0.08) | 3.07(0.09) | 0.08(0.06)* |
|  |  | 80-100 | 16(2577) | 3.65(0.07) | 2.87(0.07) | 2.58(0.09) | 0.29(0.06) |
|  |  | 100-120 | 15(262) | 3.43(0.07)* | 2.48(0.08) | 2.14(0.11) | 0.34(0.06) |
|  |  | 120-140 | 5(20) | 3.12(0.15) | 1.81(0.16) | 1.51(0.21) | 0.31(0.13) |
|  | 15-18 | 60-80 | 11(3340) | 3.95(0.08) | 3.24(0.09) | 3.04(0.11) | 0.21(0.08) |
|  |  | 80-100 | 9(873) | 3.66(0.09) | 2.78(0.10) | 2.43(0.12) | 0.35(0.07) |
|  |  | 100-120 | 5(74) | 3.52(0.10) | 2.45(0.15) | 2.09(0.17) | 0.36(0.07)* |
|  |  | 120-140 | 2(5) | 3.12(0.20) | 1.93(0.26) | 1.51(0.25) | 0.40(0.23) |

HR and HRV based on data from all 5-min segments in each recording, indices analyzed based on linear effects mixed modelling. HRV data are presented as estimated marginal means (SE). Data divided into different age groups (5-10 years, 10-15 years and 15-18 years), heart rate (HR) regions (60-80 beats per minute (bpm), 80-100 bpm, 100-120bpm, 120-140bpm) and the presence of symptoms. Age presented in years. Asymp LQTS = asymptomatic LQTS patients; Symp LQTS = symptomatic LQTS patients; HR = heart rate; Nsubj = the number of subjects in each age group; Nseg = the number of 5 minutes Holter segments; PTOT = total power; LF = power of the low frequency component; HF = power of the high frequency component; LF/HF = the ratio between the low frequency component and the high frequency component. * indicates significant differences between asymptomatic and symptomatic LQTS patients (p<0.05).

**Table S3.** Relation between heart rate and heart rate variability in boys and girls with Long QT syndrome type 1 (LQT1).

|  | Age (years) | N girls | N boys | LQTS girls | LQTS boys | LQTS girls | LQTS boys | P-value | P-value |
| --- | --- | --- | --- | --- | --- | --- | --- | --- | --- |
|  |  |  |  | Intercept | Intercept | Slope | Slope | Intercept | Slope |
| PTOT | 1-5 | 19 | 16 | 3.85 | 3.96 | -0.015 | -0.015 | 0.29 | 0.98 |
|  | 5-10 | 17 | 21 | 3.91 | 3.99 | -0.011 | -0.012 | 0.27 | 0.58 |
|  | 10-15 | 17 | 14 | 3.86 | 4 | -0.008 | -0.009 | 0.040* | 0.55 |
|  | 15-18 | 13 | 13 | 3.86 | 4.02 | -0.008 | -0.009 | 0.021* | 0.78 |
| LF | 1-5 | 19 | 16 | 3.34 | 3.36 | -0.02 | -0.019 | 0.87 | 0.56 |
|  | 5-10 | 17 | 21 | 3.32 | 3.37 | -0.017 | -0.019 | 0.46 | 0.41 |
|  | 10-15 | 17 | 14 | 3.24 | 3.35 | -0.017 | -0.016 | 0.19 | 0.62 |
|  | 15-18 | 13 | 13 | 3.19 | 3.34 | -0.017 | -0.016 | 0.06 | 0.7 |
| HF | 1-5 | 19 | 16 | 3.71 | 3.86 | -0.031 | -0.032 | 0.31 | 0.46 |
|  | 5-10 | 17 | 21 | 3.64 | 3.64 | -0.029 | -0.03 | 0.98 | 0.91 |
|  | 10-15 | 17 | 14 | 3.37 | 3.37 | -0.028 | -0.024 | 0.98 | 0.1 |
|  | 15-18 | 13 | 13 | 3.22 | 3.23 | -0.027 | -0.024 | 0.88 | 0.3 |
| LF/HF | 1-5 | 19 | 16 | -0.37 | -0.5 | 0.01 | 0.013 | 0.17 | 0.11 |
|  | 5-10 | 17 | 21 | -0.32 | -0.27 | 0.012 | 0.011 | 0.52 | 0.39 |
|  | 10-15 | 17 | 14 | -0.13 | -0.03 | 0.011 | 0.008 | 0.14 | 0.11 |
|  | 15-18 | 13 | 13 | -0.02 | 0.11 | 0.01 | 0.008 | 0.045* | 0.45 |

The relation between HR and HRV is based on data from all 5-min segments in each recording, indices analyzed based on linear effects mixed modelling. Intercepts are estimated values of HRV indices at HR=60 beats/min. Slopes are the estimated linear change in HRV indices with increasing HR. Values are based on log-10 transformed HRV expressed in ms^2^ and HR expressed in beats/min. N girls = number of girls included; N boys = number of boys included; LQTS = Long QT Syndrome; PTOT = Total power; LF = power of the low frequency component; HF = power of the high frequency component; LF/HF= ratio between the low frequency component and the high frequency component. * indicates significant differences.

**Table S4.** Relation between heart rate and heart rate variability in patients with Long QT syndrome (LQTS) with recordings both off and on betablocker treatment (N=37) and controls (N=69).

| **Intercept** | **LQTS offBB** | **LQTS onBB** | **Controls** | **p-value  offBB vs onBB** | **p-value  offBB vs Controls** |
| --- | --- | --- | --- | --- | --- |
| PTOT | 3.95 (0.05) | 3.97 (0.06) | 3.90 (0.10) | 0.18 | 0.35 |
| LF | 3.39 (0.05) | 3.34 (0.06) | 3.34 (0.11) | <0.001* | 0.38 |
| HF | 3.76 (0.07) | 3.68 (0.08) | 3.47 (0.15) | <0.001* | <0.001* |
| LF/HF | -0.37 (0.06) | -0.35 (0.07) | -0.14 (0.14) | 0.08 | 0.002* |
| **Slope** | **LQTS offBB** | **LQTS onBB** | **Controls** | **p-value  offBB vs onBB** | **p-value  offBB vs Controls** |
| PTOT | -0.012 (0.001) | -0.013 (0.001) | -0.006 (0.002) | 0.006* | <0.001* |
| LF | -0.017 (0.001) | -0.019 (0.001) | -0.012 (0.002) | <0.001* | <0.001* |
| HF | -0.028 (0.001) | -0.031 (0.001) | -0.020 (0.002) | <0.001* | <0.001* |
| LF/HF | 0.012 (0.001) | 0.012 (0.001) | 0.009 (0.002) | 0.009* | 0.01* |

The relation between HR and HRV is based on data from all 5-min segments in each recording, indices analyzed based on linear effects mixed modelling. Intercepts are estimated values of HRV indices at HR=60 beats/min. Slopes are the estimated linear change in HRV indices with increasing HR. Values are based on log-10 transformed HRV expressed in ms^2^ and HR expressed in beats/min. PTOT = total power; LF = power of the low frequency component; HF = power of the high frequency component; LF/HF = the ratio between the low frequency component and the high frequency component. * indicates significant differences.

**Figure S1**. Age-related development in heart rate variability in different heart rate regions in patients with Long QT syndrome type 1 and controls. Thin black lines show the estimated quadratic regression lines in controls (mean and 95% prediction intervals). Thick blue lines show the corresponding regression line in patients with LQT1. A linear regression model was used for LF/HF. The dots indicate the age where the maximum value of the estimated age-dependency occur. Data divided into different heart rate (HR) regions (60-80 beats per minute (bpm), 80-100 bpm, 100-120bpm, 120-140bpm). LQT1 = Long QT syndrome type 1; PTOT = total power; LF = power of the low frequency component; HF = power of the high frequency component; LF/HF = the ratio between the low frequency component and the high frequency component.
